# Functional epitopes and neutralizing antibodies of vaccinia virus

**DOI:** 10.3389/fmicb.2023.1255935

**Published:** 2023-10-25

**Authors:** Fenghao Peng, Naijing Hu, Yingjun Liu, Cong Xing, Longlong Luo, Xinying Li, Jing Wang, Guojiang Chen, He Xiao, Chenghua Liu, Beifen Shen, Jiannan Feng, Chunxia Qiao

**Affiliations:** ^1^State Key Laboratory of Toxicology and Medical Countermeasures, Institute of Pharmacology and Toxicology, Beijing, China; ^2^School of Medicine and Holistic Integrative Medicine, Nanjing University of Chinese Medicine, Nanjing, China; ^3^Joint National Laboratory for Antibody Drug Engineering, The First Affiliated Hospital, School of Medicine, Henan University, Kaifeng, China

**Keywords:** vaccinia virus, variola virus, epitope, neutralizing antibody, engineered antibody

## Abstract

Smallpox is an infectious disease caused by the variola virus, and it has a high mortality rate. Historically it has broken out in many countries and it was a great threat to human health. Smallpox was declared eradicated in 1980, and Many countries stopped nation-wide smallpox vaccinations at that time. In recent years the potential threat of bioterrorism using smallpox has led to resumed research on the treatment and prevention of smallpox. Effective ways of preventing and treating smallpox infection have been reported, including vaccination, chemical drugs, neutralizing antibodies, and clinical symptomatic therapies. Antibody treatments include anti-sera, murine monoclonal antibodies, and engineered humanized or human antibodies. Engineered antibodies are homologous, safe, and effective. The development of humanized and genetically engineered antibodies against variola virus via molecular biology and bioinformatics is therefore a potentially fruitful prospect with respect to field application. Natural smallpox virus is inaccessible, therefore most research about prevention and/or treatment of smallpox were done using vaccinia virus, which is much safer and highly homologous to smallpox. Herein we summarize vaccinia virus epitope information reported to date, and discuss neutralizing antibodies with potential value for field application.

## 1. Introduction

Variola virus (the smallpox virus) is one of the largest and most complex viruses in the world. It is a member of the genus *Orthopoxvirus* of the *Chordopoxvirinae* subfamily of the *Poxviridae* family (Theves et al., 2016). Smallpox is a highly contagious virus that only infects humans. There are two forms of infectious viral particles; mature virions (MV) and enveloped virions (EV). MVs are the main form, and they play a major role in the spread of the virus between hosts. EV is formed by an MV and an extracellular enveloped membrane. By experimental operation, the enveloped membrane of EV is easy to be destroyed (Condit et al., 2006; Roberts and Smith, 2008). EV facilitates infection between cells (Roberts and Smith, 2008).

Poxviruses include a large family of viruses characterized by large linear dsDNA genomes, cytoplasmic replication sites, and complex virion morphology (Lefkowitz et al., 2005; Condit et al., 2006). The prototype laboratory virus used for poxvirus research was vaccinia virus, which was used as a live, naturally attenuated vaccine to eradicate smallpox (Roberts and Smith, 2008). Members of the poxvirus family are very similar, so lessons learned from vaccinia can easily be applied to other poxviruses. Vaccinia virus particles are “brick-like” or “ovoid” membrane-bound particles with a complex internal structure characterized by a walled, double-concave core flanked by “lateral bodies.” VACV produces two different forms of infectious virion, both of which are targets of antibody responses to smallpox vaccine. Most infectious VACVs are intracellular MV, which remain inside the cell until cell lysis. MV has a membrane that is associated with at least 19 different viral proteins. A27, L1, D8, H3, and A28 are known targets of neutralizing antibodies. A small portion of the MV in the cell gains additional membrane by being wrapped in the Golgi cisternae. They are eventually released as EV through exocytosis and are responsible for the virus’s long-distance transmission within the host. EV has an additional outer membrane than MV and is associated with at least six different viral proteins, with B5 being the primary target for neutralizing antibodies and A33 triggering a protective antibody response. For optimal smallpox immune protection, antibodies against both smallpox virus MV and EV are required (Roberts and Smith, 2008). Antibodies block the transmission of the virus between and within individuals by recognizing epitopes of MV and EV.

The mortality rate of smallpox is >50% (Fulginiti et al., 2003; Nafziger, 2005). Starting in the 17th century smallpox caused a worldwide pandemic that killed approximately 400,000 people every year in Europe, and blinded one third of those it infected. In the 20th century smallpox killed at least 300 million people worldwide. From 1967 to the end of the 1970s a widespread campaign aimed at elimination via vaccination was implemented, resulting in the eradication of smallpox. In 1980 the World Health Organization (WHO) announced the eradication of smallpox, and ceased worldwide vaccination against the disease. Currently only two secure laboratories, one in Russia and one in the US, are authorized to store live smallpox virus.

Historically smallpox has never been used as a weapon in wars because of its high infectivity and lethality. Now, the smallpox virus has become one of the best materials for use in biological warfare or bioterrorism (Pennington, 2003). After the 9/11 attack in the US the smallpox virus was listed as one of the most important biological agents with potential use for terrorist attacks in that country. The cessation of smallpox immunization means a large number of people today have no resistance against smallpox. Other *Orthopoxvirus* spp. and their possible mutation or recombination in nature may also threaten human health. Thus, it is necessary to research potential antagonists against smallpox.

Vaccination is a simple and effective means of preventing smallpox before or after exposure to the virus (Mayr, 2003; Meyer et al., 2020). Expanding the reserves of smallpox vaccines has become a global need. Although the efficacy of traditional smallpox vaccines has been fully verified they sometimes did cause side effects (Parrino and Graham, 2006), which necessitates the development of a safer and more effective smallpox vaccine. Several different types of smallpox vaccines have been developed, including cell-cultured live virus vaccines, replicating and non-replicating attenuated live virus vaccines, protein-based subunit vaccines, DNA-based subunit vaccines, and vector-based subunit vaccines (Addeo et al., 2021). Dryvax (Amanna et al., 2006) is a traditional smallpox vaccine that is no longer stored in the United States. Currently the two main vaccines are ACAM2000 (Beachkofsky et al., 2010; Nalca and Zumbrun, 2010) and Jynneos (Kennedy and Greenberg, 2009; Rao et al., 2022). In the event of a smallpox outbreak, antiviral drugs such as cidofovir, ribavirin, and tecovirimat (TPOXX®; ST-246) (Russo et al., 2021) could be used for emergency treatment. Tecovirimat is a potent antiviral that was approved for the treatment of symptomatic smallpox by the US Food and Drug Administration in July 2018, and it has been stockpiled by the US government for use in a smallpox outbreak.

Antibodies have been used as therapeutic agents for hundreds of years, including antiserum, mouse monoclonal antibodies, and human/humanized antibodies. In individuals for whom smallpox vaccination is contraindicated, specific vaccinia immunoglobulin (VIG) or monoclonal antibodies (mAbs) are alternative strategies for preventive or post-infection treatment. The indications that grant the use of VIG include generalized vaccinia, progressive vaccinia, eczema vaccinatum, and certain accidental implantations. Data suggest VIG efficacy for prophylaxis of vaccinial superinfection of eczema, burns, chickenpox, immunosuppression, pregnancy, or certain skin conditions (Hopkins and Lane, 2004). There is limited potential for the broadscale use of VIG extracted from the serum of vaccine recipients however (Hopkins and Lane, 2004), and VIG obtained from animals such as mice or rabbits tends to cause side effects. Murine mAbs also have several disadvantages in humans, including a lack of antibody-dependent cell-mediated cytotoxicity, complement-dependent cytotoxicity, a short half-life, and human anti-mouse antibody reactions, which reduce efficacy and can cause allergic reactions (Hansel et al., 2010). Therefore the development of humanized and genetically engineered antibodies against smallpox virus via molecular biology, immunology, and bioinformatic methods is a worthwhile prospect. Such endeavors require an understanding of the antigenic epitopes of smallpox viral proteins (e.g., A27, B5, D8) to facilitate the generation of neutralizing antibodies.

Because the smallpox virus is dangerous and currently stored under highly regulated conditions, the effects of anti-smallpox antibodies are estimated using the vaccinia virus (VACV). Most research about prevention and/or treatment of smallpox were done using vaccinia virus, which is much safer and highly homologous to smallpox. At least 20 proteins have been identified on the surface of smallpox MVs, and 6 have been identified on EV. Rodriguez et al. (1985) isolated a series of monoclonal antibodies against VACV, including anti-B5 monoclonal antibody MAb20 and anti-A27 monoclonal antibody C3. In 2011 Meng et al. (2011) immunized BALB/c mice with VACV, and after fusion and clone screening 66 mAbs were obtained. Their epitopes were identified on 11 proteins; D8, A14, wRl48, D13, H3, A56, A33, C3, B5, A10, and F13. The proteins recognized by neutralizing antibodies include A33 (Fogg et al., 2004; Fang et al., 2006), B5 (Fogg et al., 2004; Aldaz-Carroll et al., 2007), L1 (Wolffe et al., 1995; Ichihashi and Oie, 1996; Fogg et al., 2004), H3 (Lin et al., 2000; Davies et al., 2005), A27, D8 (Sakhatskyy et al., 2006), A28 (Nelson et al., 2008), A17 (Wallengren et al., 2001), A30, B7, and F8. Among them, L1 (Bisht et al., 2008), H3 (Lin et al., 2000), A27 (Chung et al., 1998; Vázquez and Esteban, 1999), D8 (Hsiao et al., 1999), and A28 (Senkevich et al., 2004) are related to virus adsorption, membrane fusion, and virus entry. B5 is involved in virus packaging, particle release, virus morphology, plaque formation, and intercellular infection (Aldaz-Carroll et al., 2005). A33 is related to virus transmission between cells. Proteins with neutralizing epitopes include the EV proteins B5, A33 (Breiman and Smith, 2010; Breiman et al., 2013; Monticelli et al., 2020), and the MV proteins H3, L1, D8, A13, A27, A17, and A28. Those proteins and their monoclonal antibodies are reviewed below.

## 2. Viral proteins and protective mAbs

### 2.1. B5

B5 is an EV protein that is highly conserved among orthopoxviruses (Engelstad and Smith, 1993). It is a glycosylated type I membrane protein with a relative molecular weight of 4.2 × 10^4^ Da. The extracellular domain of B5 contains four short repeat domains similar to complement regulatory proteins, but it has no notable complementary function. B5 is necessary for virus packaging, and it contains epitopes that are recognized by neutralizing antibodies which block virus infection (Bell et al., 2004; Aldaz-Carroll et al., 2007; Benhnia et al., 2009), providing a protective effect both *in vitro* and *in vivo*. The localization of B5 to the surface of intracellular EV and its transport from the endoplasmic reticulum to the Golgi network is dependent on its interaction with A33 and A34. A33, A34, and B5 form a trimeric protein complex that is vital for their endoplasmic reticulum exit and Golgi transportation. The three glycoproteins efficiently localize and become incorporated into the outer extracellular virion membrane, and directly influence the release of infectious poxvirions (Monticelli et al., 2020).

Chen et al. (2006) generated two human anti-B5 mAbs via phage display technology, 8AH7AL and 8AH8AL. The two mAbs displayed high binding affinities to B5. In a mouse lung infection model administration of 22.5 μg 8AH8AL 24 h prior to infection provided complete protection, and 5 mg VIG provided similar protection against intranasal inoculation with 10^5^ plaque-forming units (PFU) of VACV (WR strain). Administration of 90 μg 8AH8AL 48 h post-infection also completely protected mice. The mAb 8AH8AL inhibited the spread of vaccinia virus *in vitro*, protected mice from subsequent intranasal challenge with virulent vaccinia virus. However, 5 mg VIG sometimes led to death, indicating a worse therapeutic effect than that of 8AH8AL. Froude et al. (2011) generated a humanized anti-B5 hB5R mAb whose maternal antibody was isolated from immunized rats. In a murine model where mice were infected with 10^7^ VACV PFU intranasally and given 10 μg of antibody intraperitoneally 5 h later, body weight loss was reduced relative to the control group.

### 2.2. A33

A33 is a specific EV type II membrane glycoprotein involved in the efficient formation of endocellular enveloped virus, and transmission of the virus in the host (Matho et al., 2015). It has strong immunogenicity and effectively induces protective antibodies *in vivo*. Pre-immunization with vaccines containing the A33 subunit or post-treatment with anti-A33 antibodies may help protect animals infected with a lethal dose of VACV (Galmiche et al., 1999; Zajonc, 2017). In *in vitro* models anti-A33 antibodies inhibited comet formation, suggesting they can block intercellular transmission (Galmiche et al., 1999).

Three fragment antigen-binding (Fab) regions that recognize overlapped epitopes of A33 glycoprotein have been isolated from simians; 6C, 12F, and 12C (Chen et al., 2007). The corresponding genes were fused with the human heavy chain constant region to form full antibodies. Their affinities were 20 nmol/L (6C), 0.14 nmol/L (12F), and 0.46 nmol/L (12C). The affinity of 6C was approximately 140 times weaker than that of 12F, but its neutralizing effects were similar to those of 12F both *in vitro* and *in vivo*. In a mouse model of intranasally introduced infection, injection of 6C 24 h before infection completely protected mice from death and minimized weight loss, and weight loss recovery was observed within 15 d post-infection. As little as 22.5 μg of 6C could completely protect mice to a similar degree as 5 mg of VIG. In addition, whether mice were injected 24 h before or 48 h after infection, 90 μg 6C or the anti-B5 mAb 8AH8AL—or a combination of both (45 μg each)—completely protected mice from death. Anti-B5 mAb-treated mice exhibited less weight loss during 15 d of treatment, particularly in the post-infection administration experiment, amounting to better efficacy than that demonstrated by 6C. The efficacy of the combination group was between that of the two single antibody groups.

Matho et al. (2015) generated seven mouse anti-A33 mAbs that bind to conformational epitopes on A33 rather than linear epitopes, five of which neutralized endocellular enveloped virus in the presence of complement. The authors elucidated the crystal structures of three representative neutralizing mAbs (A2C7, A20G2, and A27D7), then estimated the binding kinetics of each to wild-type A33 and to an engineered A33 protein containing a single alanine substitution in the epitope area. A2C7 and A20G2 are bound to a single A33 subunit, whereas A27D7 is bound to both A33 subunits. Alanine substitution did not affect the binding of A27D7, which also showed high affinity binding to the recombinant A33 protein. A27D7 was an effective cross-neutralizer against orthopoxvirus strains such as agamia virus, monkeypox virus, and VACV, and it protected mice from lethal challenge with agamia virus (Matho et al., 2015).

Paran et al. (2013) reported that a single dose of Sindbis VACV A33 (a recombinant vaccinia virus protein A33 using Sindbis virus-expressing System) did not protect mice from cowpox virus infection, but effectively protected mice from VACV-WR and ectromelia virus challenges. Homologous vaccination with cowpox virus A33 also failed to protect mice from cowpox challenge, and provided only partial protection against VACV-WR. A single protective region located in residues 104–120 of VACV A33 was identified that carries the H2Kd CD8^+^ T cell epitope and the B cell epitope, recognized by the neutralizing antibody mAb 1G10, which effectively blocks extracellular virion transmission (Paran et al., 2013).

Mucker et al. (2020) evaluated the ability of the anti-A33 humanized monoclonal antibody C6C to affect VACV infection *in vitro*. Enveloped virions released from infected cells were either sensitive or resistant to C6C, suggesting that different types of biologically distinct extracellular virions particles exist, including extracellular enveloped virions and cell-associated released virions. In addition, mAb C6C bound to the recombinant A33 homolog of the Zaire strain of the monkeypox virus (Mucker et al., 2020).

### 2.3. L1

L1 is an MV membrane protein with a relative molecular mass of 2.9 × 10^4^ Da that is highly conserved among all sequenced poxviruses. It has a transmembrane domain at the C-terminus, and no signal peptide at the N-terminus, but it is myristoylated and can attach to the membrane easily. The L1 protein is required for the virus to enter host cells, and is an important target recognized by neutralizing antibodies. It was originally identified using the neutralizing antibody 7D11 (Wolffe et al., 1995). L1 is comprised of three pairs of disulfide bonds, two of which are necessary for the production of infectious virions (Blouch et al., 2005). Once the disulfide bonds are broken, L1 is not recognized by neutralizing antibodies (Wolffe et al., 1995; Ichihashi and Oie, 1996). Ichihashi et al. (1994) produced the anti-L1 neutralizing antibodies 2D5 and 8C2, which block viral cell plaque formation. Su et al. (2007) investigated the neutralizing effects of the mouse anti-L1 monoclonal antibody 7D11, including its Fab, F (ab’), and full IgG using plaque formation assays, and reported that the neutralizing effects of Fab were weaker. X-ray diffraction techniques revealed the crystal structure of 7D11 interacting with the L1 protein. Based on the contact area and inter-molecular distance, 7D11 bound to the L1 antigen mainly via its heavy chain, and the effect of the light chain was very weak. 7D11 also formed hydrogen bonds and van der Waals interactions with loop l and loop 2 of L1. 7D11 binding sites are conserved among VACV, smallpox virus, and monkeypox virus suggesting that 7D11 may exhibit cross-protective effects. Kaever et al. (2014) generated five mouse anti-L1 mAbs, which can be categorized into three groups based on their epitopes. At a concentration of 20 g/mL three mAbs (M12B9, M2E9, and M7B6) neutralized more than 70% of VACV, but the other two did not neutralize the virus. The neutralizing antibodies have a higher affinity for the recombinant L1 protein than the non-neutralizing antibodies, and they also bind to viral particles. The epitopes of the neutralizing antibodies were mapped to a conformational epitope with Asp35 as the key residue, and the epitope was similar to that of 7D11 (Kaever et al., 2014). By immunizing two alpacas Walper et al. (2014) generated multiple specific single-domain antibodies with affinities ranging from 4 × 10^−9^ M to 7 × 10^−10^ M. The single-domain antibodies, as capture and tracer agents, reduced the detection limit to 4 × 10^5^ PFU/mL in a sandwich assay–a four-fold improvement over conventional antibodies. This demonstrates the development of single-domain antibodies and the ability to detect viruses in sandwich assays (Walper et al., 2014).

### 2.4. D8

Matho et al. (2014) described the crystal structure of the adhesion protein D8. Its N-terminal domain contains a carbonic anhydrase fold region (CAH; residues 1–234) followed by a smaller domain (residues 235–273). The remainder of the protein consists of a transmembrane domain (274–294) and a small tail (295–304) within the virion. The CAH domain can bind to glycosaminoglycans and chondroitin sulfate (CS) in host cells because it has a central positively charged gap that complements the negative charge of CS. The optimal ligand for D8 is CS-E, which is characterized as a disaccharide moiety with two sulfate hydroxyl groups at the 4′ and 6′ positions of GalNAc (Matho et al., 2014). Hsiao et al. (1999) constructed A27 and D8 single null, and A27-D8 double-null virus strains based on the WR32-7/Indl4K virus strain. The A27-null virus was amplified in BSC40 cells, but the infectivity of the D8 null and A27-D8 double null strains was significantly lower than that of the wild-type virus, with virulence of only 10%. This indicates that D8 is key for virus infection and endocytosis, and that the A27 protein cannot compensate for loss of D8 function.

Sakhatskyy et al. (2006) constructed a DNA vaccine encoding D8 and immunized BALB/c mice, which induced neutralizing antiserum and protected mice against a lethal dose of VACV. Addition of the D8 protein to the existing subunit vaccine induced antibodies with better neutralizing activity. Based on this, they proposed that D8 was a satisfactory recognition target for neutralizing antibodies. Matho et al. (2012) generated the anti-D8 monoclonal antibody LA5, which is capable of neutralizing VACV in the presence of complement. They described the D8 and LA5 Fab structures to respective resolutions of 0.142 and 0.16 nm, and the crystal structure of the LA5 Fab-D8 complex to 0.21 nm. Based on these structures they predicted that the binding site of CS is located in the central positively charged gap of the D8 molecule. The structure of the gap is highly conserved across several poxviruses. The D8 epitope recognized by LA5 consists of 23 discrete residues scattered across 80% of the D8 sequence. Interestingly LA5 binds to the region above the gap with high affinity, and the antigen–antibody interaction area is unusually large, covering the 243.4 nm protein surface.

Matho et al. tested the capacity of a panel of mouse monoclonal antibodies to compete with CS-E for D8 binding. CS-E binding was only completely abolished by LA5. D8 forms a hexameric arrangement via the self-association of its C-terminal domain. Oligomerization of D8 allows VACV to adhere to multiple CS variants, including CS-C and potentially CS-A, thus improving overall binding efficiency to CS-E (Matho et al., 2014). Matho et al. characterized several epitopes of human D8 antibodies (VACV66, VACV-138, and VACV-304) and determined the first crystal structures of human antibodies that bind to D8. The epitopes are located in the CAH domain, which possesses moderate neutralizing activity in the presence of complement. The crystal structures of VACV-66, VACV-138, and VACV-304 bound to the D8 CAH domain have respective resolutions of 2.23, 2.90, and 2.90 Å. VACV-138 and VACV-304 completely block the binding of D8 to CS-A, whereas VACV-304 only partially blocks binding of D8 to the high-affinity ligand CS-E, indicating the presence of both a high-affinity and a low-affinity CS binding region in the D8 gap. VACV-66 laterally binds to D8 far away from the CS-binding gap, explaining why VACV-66 does not interfere with D8 binding to CS-E (Matho et al., 2018).

### 2.5. A13

The theoretical molecular weight of A13 is 8 × 10^3^ Da, but some studies show that A13 migrates to 1.2 × 10^4^ Da in SDS-PAGE analysis. A13 is an antigenic molecule recognized by neutralizing antibodies. Xu et al. (2011) described the mAb 11F7 (IgG2a), which bound to A13 with an affinity of 3.4 nM/L and neutralized MVs. The antibody recognizes the 10-amino acid epitope ISSLYNLVKSS which is highly conserved among *Orthopoxvirus* species, including VACV and monkeypox virus, indicating its potential to exert a wide range of protective effects. BALB/c mice were injected intraperitoneally with 2 mg of the antibody 24 h before intranasal challenge with VACV WR virus, and changes in body weight and mortality rate were observed. After challenged with VACV WR, all mice lost significant body weight, but mice that received either 11F7 or anti-H3 #41 lost less weight on average than mice that received PBS. mice that received anti-B5 antibody B126 lost less weight on average than mice that received 11F7. In addition, more mice that received B126 (100%), 11F7 (80%) or anti-H3 (80%) survived the challenge than mice that received PBS (40%). Anti-A13 antibody had good protective effects, and the efficacy of anti-A13 antibody alone was similar to that of anti-H3 antibody #41. The therapeutic effect of the anti-A13 antibody is evidently weaker than that of the anti-B5 antibody B126 (IgG2a) (Benhnia et al., 2009) administered orally. Mice administered B126 alone and in combination with anti-A13 survived and maintained body weight, and body weight in the combined group was greater than that in the B126 alone group.

### 2.6. H3

H3 is the envelope protein of MV and has a relative molecular mass of 3.5 × 10^4^ Da. The gene encoding H3 is a late gene in the MV virus, and its sequence is highly conserved among members of the poxvirus family. H3 can bind to heparan sulfate on the cell surface, which is related to the adsorption of MV to cells. Lin et al. (2000) constructed an H3-deficient virus that had a smaller plaque size, a virulence one tenth that of the wild-type virus, and a modified morphology. Notably however, H3 is not involved in cell fusion. In a mouse model involving intranasal virus inoculation, mice inoculated with wild-type virus had higher mortality and greater weight loss than mice infected with the H3-deficient virus, which exhibited a higher survival rate and faster recovery. These observations indicated that the H3 protein is related to virus infection, and that the toxicity of H3-deficient virus *in vivo* is reduced. The H3 protein evidently also plays a role in the assembly of virus particles. Davies et al. (2005) reported that H3-specific antibodies are detectable in most people vaccinated with the Dryvax vaccine, especially after a second vaccination. Anti-H3 polyclonal antibody purified from human serum was able to reduce plaque formation by 50% at a dose of 44 μg/mL. After mice were immunized with Dryvax, anti-H3 antibodies were detected in serum via protein microarray technology. Mice further immunized with H3 had serum neutralizing activity higher than that obtained with the VV_NYBOH_ vaccine strain (anti-H3 antiserum had a functional titer of 1:3760, whereas anti-vaccine antiserum had a functional titer of 1:172). Moreover, immunized mice resisted challenges with intranasally administered VACV WR as high as 5 × LD_50_. In passive transfer experiments using anti-H3 antiserum some mice were able to resist a challenge with 3 × LD_50_ VACV WR (survival rates were 5/10 in the antiserum group and 0/10 in the control group, *p* < 0.05).

### 2.7. A27

A27 is another MV membrane protein that can bind glycosaminoglycans on the cell surface and mediate the fusion of virus and cell. The A27 protein is a trimer containing two parallel α-helices and one antiparallel α-helix (Wang et al., 2014). The structure of A27 is similar to that of influenza hemagglutinin or HIV gp41, except A27 has no membrane-anchoring sequence. Instead it has a domain that interacts with the A17 protein, thus A17 is considered to be a membrane-anchoring helper for A27. The C-terminus of A27 interacts specifically with the N-terminus of A17 via a parallel, cooperative binding mechanism at the F1 and F2 binding sites. Thr88-Lys99 of A27 interacts with Ser32-Lys36 of A17 at the F1 binding site, and Phe80-Glu87 of A27 binds to Leu20-Gln29 of A17 at the F2 binding site (Wang et al., 2014). A27 and A26 form a stable complex, and this helps A17 to bind to the surface of MV particles (Howard et al., 2008).

He et al. (2007) tested the neutralizing titer of anti-A27 antibodies in antiserum after Dryvax vaccination. Antibodies binding to recombinant A27 protein were detected in the antiserum, but neutralizing capacity was not significantly weakened after removal of A27 antibodies. Antibodies against recombinant A27 protein were used in passive transfer experiments, and they enhanced the neutralizing capacity of VIG, indicating that A27 is a neutralizing epitope. A27 is not the main epitope recognized by VIG however, at least in Dryvax vaccine antiserum. Fogg et al. (2008) reported that the antibodies produced by A27-immunized and L1-immunized mice were comparable. In an intranasal virus mouse model however, the anti-A27 antibody was less effective than the anti-L1 antibody. In addition, the ability of mice immunized with both L1 and A33 to resist virus infection was worse than that of animals immunized with A27 and A33. In cytological experiments rabbit anti-L1 polyclonal antibody had a comparable neutralizing effect to anti-A27 polyclonal antibody in both human and mouse cell lines, and in glycosaminoglycan-deficient cell lines, irrespective of whether the antibody was given before or after virus adsorption. This suggests that early identification of neutralizing antibodies is necessary in animal models, at least for the identification of A27 and L1 antibodies.

Thomas et al. produced and characterized three groups of mAbs. All group I mAbs (1G6, 12G2, and 8H10) bound to a linear peptide spanning residues 21–40, located near the glycosaminoglycan binding site of A27. These mAbs could neutralize MV and resist VACV attack in a complement-dependent manner. This suggests that the group I mAbs may interfere with A27 cell adhesion. The crystal structures of 1G6 and the non-neutralizing mAb 8E3 bound to the corresponding linear epitope-containing peptides indicate that both the light and heavy chains of the antibody are important for binding to the antigen. For both antibodies, the L1 loop is important for the overall polar interaction with the antigen, whereas for 8E3 the light chain was more important for contact with the antigen. mAbs that bound to the functional region of antigens (e.g., mAb 1G6) provided greater protection than those that bound to the distal region (e.g., mAb 8E3) (Kaever et al., 2016).

### 2.8. A17

The precursor of A17 has a relative molecular mass of 2.3 × 10^4^ Da and contains 203 amino acids. It is hydrolyzed by protease at the AA17 site to obtain the A17 protein. A17 has two hydrophobic peptide segments, thus both its N-terminus and C-terminus were once thought to be located inside the membrane to act as a membrane-anchoring helper of the A27 molecule, not as a membrane protein and antibody recognition epitope of MV. Wallengren et al. (2001) identified a series of rabbit polyclonal antibodies against different segments of A17 via immunoelectron microscopy, immunoblotting, and neutralization assays in BSC-40 cells. Their investigations indicated that the polyclonal antibodies against the C-terminus of the A17 protein had no protective effects, whereas anti-N-terminus and anti-A17 protein antibodies had good protective effects. These observations suggested that the extracellular N-terminus of A17 contained the neutralizing epitopes, whereas the C-terminus was located inside the MV membrane and did not induce neutralizing antiserum.

### 2.9. A28

The poxvirus cell/membrane complex consists of at least nine transmembrane proteins (including A28 and H2) that are conserved among all poxviruses, although the physical structure and immunogenicity of each component are not well understood. Nelson et al. (2008) expressed and purified soluble A28 protein in an insect expression system and generated rabbit anti-A28 polyclonal antibody. This antibody neutralized VACV and prevented it from entering cells. In an *in vivo* intranasal inoculation mouse model the virus caused significant weight loss that was inhibited by administration of the polyclonal antibody. Its neutralizing effects were similar to those exerted against VACV; however, the anti-H2 polyclonal antibody had no neutralizing effects. ELISA results derived from peptides of 20 amino acids in length designed based on A28 indicated that the polyclonal antibody is recognized mainly at the C-terminal amino acids, encompassing approximately one third of the total length. Antibodies binding to each peptide were obtained by affinity purification, and the antibody recognizing residues 73–92 of A28 had the best neutralizing activity (EC_50_ was 0.11 μg/mL); which was better than that of the original polyclonal antibody. The activity of other antibodies was similar to or worse than that of the polyclonal antibody, indicating that the sequence is key to epitope recognition. Shinoda et al. (2010) investigated the effects interaction between A28 and H2 on the production of anti-A28 neutralizing antibodies. Higher titers of antibodies were obtained with simultaneous immunization of A28 and H2 genes, and neutralizing activity *in vitro* and *in vivo* was stronger than that obtained via a single immunization with either A28 or H2, or even anti-A28 antiserum mixed with anti-H2 antiserum. This suggests that on the virus surface, interaction between H2 and A28 can stabilize the conformation of A28. Thus the epitope recognized by the anti-A28 antibody is mainly located at the C-terminal of A28, consistent with Nelson’s above-described study.

## 3. Multivalent antibodies and recombinant polyclonal antibodies

The body produces billions of antibodies against different antigens and different antigenic epitopes of the same antigen. Isolated antiserum has a good curative effect that is typically better than some mAbs, particularly when the antigenic epitopes are mutated. Antiserum also has obvious shortcomings however, including heterogeneity of animal origin, low safety, low proportions of effective antibodies, limited supply, and poor batch-to-batch consistency. Antisera contain multiple neutralizing antibody components (Bell et al., 2004; Goldsmith et al., 2004; Davies et al., 2005, 2007; He et al., 2007; Benhnia et al., 2008). In vaccine studies immunization with subunit vaccines of A27/L1 (MV particles) and B5/A33 (EV particles) can protect mice and rhesus monkeys from poxvirus challenge (Hooper et al., 2003, 2004; Sakhatskyy et al., 2006). A33, B5, and L1 fusion proteins can also protect mice from lethal doses of virus infection (Fogg et al., 2004). Therefore, to obtain a similar or better therapeutic effect than that derived from antiserum, it is best to combine two or more antibodies targeting both EV and MV proteins. Two human monoclonal antibodies obtained from transgenic mice, hV26 and h101, recognized the H3 protein of MV and the B5 protein of EV, respectively (McCausland et al., 2010). A dose of 20 μg hV26 was as effective as 1.25 mg antiserum in the treatment of SCID mice. The efficacy of 50 μg h101 was similar to that of 1.25 mg antiserum, and the lowest dose of 25 μg antibody was superior to 1.25 mg VIG in terms of body weight loss and clinical score. In an *in vivo* evaluation of antibody combinations 50% of mice were protected by 50 μg mAb, 30% of mice were protected by 25 μg mAb, and all mice died in the control group and the 1.25 mg antiserum group.

With the development of human antibody library and site-specific integration technology, it is possible to generate recombinant polyclonal antibodies with all the advantages of antiserum and mAbs. Moreover, recombinant polyclonal antibodies have the advantage of batch-to-batch stability, making them the best choice for treating complex infectious diseases and cancers (Haurum and Bregenholt, 2005). The company Symphogen (Lyngby, Denmark) is currently working on recombinant polyclonal antibody drugs. The first fully human recombinant polyclonal antibody formulation, Sym001 against RhD, is composed of 25 antibodies. A phase II clinical trial was done in 2012 in which Sym001 was used for the treatment of hemolytic diseases in newborns, and congenital thrombocytopenic purpura. Several other recombinant polyclonal antibody drugs are in various stages of development. These include the anti-VACV recombinant polyclonal antibody formulation Sym002 (Haurum, 2006), anti-RSV Sym003, anti-*Pseudomonas aeruginosa* Sym006, Sym008, and Sym009 (all against infectious diseases with undisclosed targets), anti-EGFR Sym004 for tumor treatment (of which a phase II clinical trial is soon to commence), and the anti-Her family member Sym013.

## 4. Conclusion

Smallpox is a severe infectious disease caused by the variola virus. Traditional vaccinations should usually be injected before exposure to viruses, and sometimes the vaccines might have unpredictable side effects. Human-sourced antiserum supply is limited, and its anti-viral efficacy is insufficient because of the low proportion of effective antibodies (Hopkins and Lane, 2004; Wittek, 2006). Thus, the development of anti-smallpox antibodies is worthwhile. To date the development of mAbs against smallpox has yielded numerous drug candidates with good efficacy *in vivo* and *in vitro*, and notably antibody cocktails targeting multiple epitopes have proven more effective than monoclonal antibodies alone. Given epitope escape caused by virus mutation, the development of multivalent antibody drugs capable of recognizing multiple epitopes will be beneficial for treating viral infections. The neutralizing epitopes of vaccinia virus reviewed herein could be used as candidate fragments for epitope combinations. Moreover, we suggest that epitope combinations should include both EV and MV proteins, such as those targeted by anti-B5 and anti-L1 antibodies, to better block the transmission of the virus between and within individuals. With continued research, new neutralizing epitopes may be discovered. The study of epitopes, their associated mechanisms and antigenicity, and their application has important practical significance with respect to preparing for potential bioterrorism involving the smallpox virus, and with regard to preventing and treating similar infectious pathogens such as severe acute respiratory syndrome viruses, H1N1 and H5N1 influenza strains, and West Nile virus.

## Author contributions

FP: Writing – original draft, Writing – review & editing. NH: Writing – original draft, Writing – review & editing. YL: Writing – original draft. CX: Writing – original draft. LL: Writing – review & editing. XL: Writing – review & editing. JW: Writing – review & editing. GC: Writing – review & editing. HX: Writing – review & editing. CL: Writing – review & editing. BS: Writing – original draft, Writing – review & editing. JF: Writing – original draft, Writing – review & editing. CQ: Writing – original draft, Writing – review & editing.
